# Supplementary material for: Assessing the integrity of auditory sensory memory processing in CLN3 disease (Juvenile Neuronal Ceroid Lipofuscinosis (Batten disease)): an auditory evoked potential study of the duration-evoked mismatch negativity (MMN)
Source: J Neurodev Disord. 2024 Jan 6;16:3. doi: 10.1186/s11689-023-09515-8 (PMC10770910; doi:10.1186/s11689-023-09515-8)
Supplement: Supplementary file 1 — Additional file 1. [file 11689_2023_9515_MOESM1_ESM.docx]

**Supplementary figures and tables:**

**
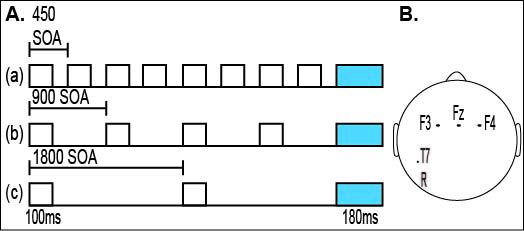
**

**Supplementary Figure 1.** Oddball paradigm design. A. Three experimental conditions were implemented, in which auditory stimuli were presented with varied stimulus onset asynchronies (SOAs): (a) 450 ms intervals between stimuli, (b) 900 ms interval between stimuli and (c) 1800 ms intervals between stimuli. B. The electrode sites of interest (F3, Fz, F4) and reference electrode R (T7) are shown on a head map.


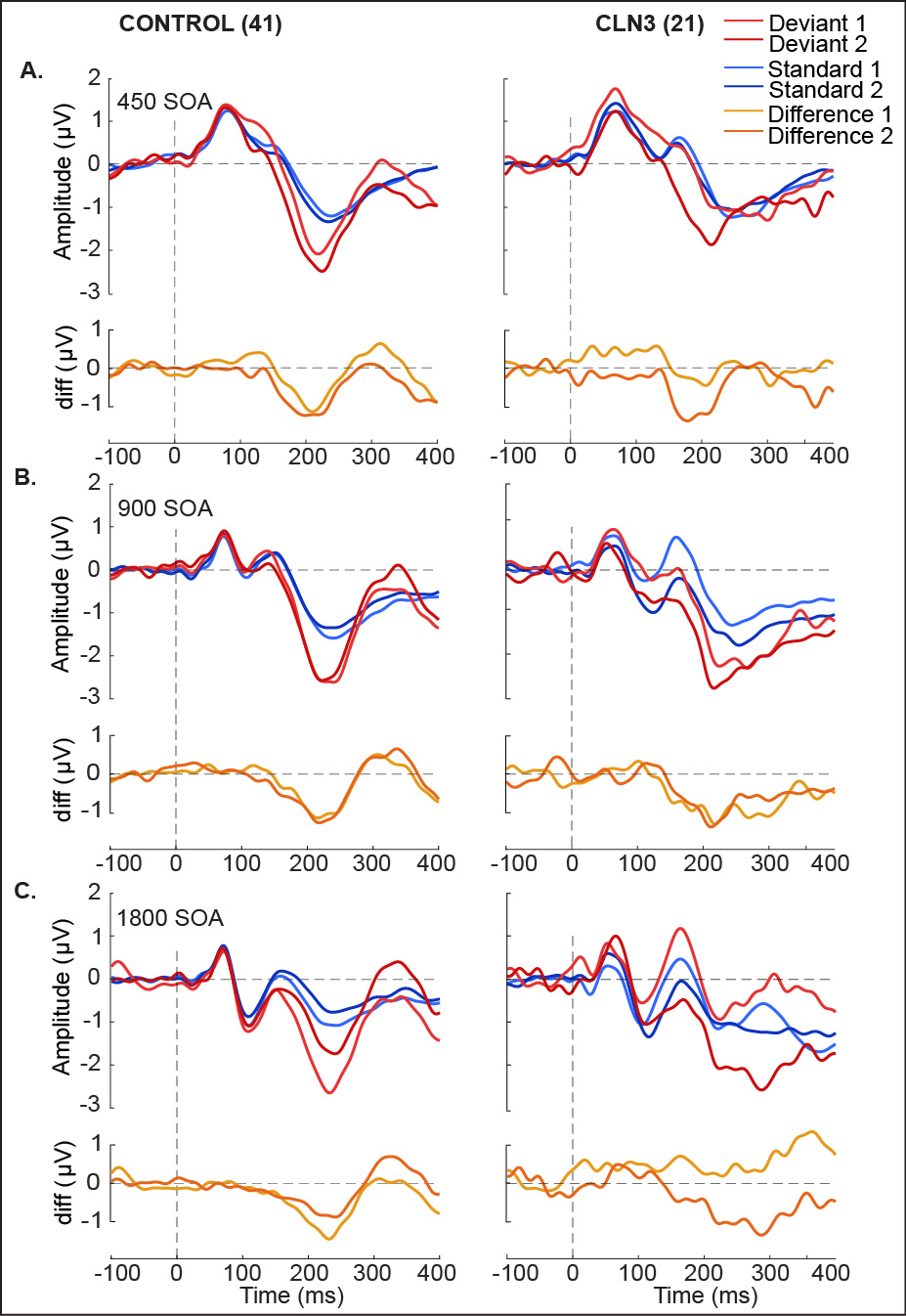


**Supplementary Figure 2.** **Half-split group-averaged waveforms.** First half versus second half of the experiment in typically developing (TD) controls and in individuals with CLN3 disease over frontal scalp sites (composite average of F3, Fz and F4).  Event-related potentials (ERPs) to standard tones (light blue trace – Standard 1 represents the first half and the dark blue trace – Standard 2 represents the second half of the experiment) and deviant tones (light red trace – Deviant 1 represents the first half and the dark red trace represents the second half of the experiment) are presented. Stimulus onset was at 0ms, indicated by the vertical dotted line. Panel A shows responses for the fastest stimulation rate (450ms stimulus onset asynchrony (SOA)). Plotted in the panel below the ERPs is the subtraction waveform (deviant minus standard), isolating the MMN-related activity (yellow trace – Difference 1 represents the first half and the orange trace represents the second half of the experiment). Despite the lower number of trials in each condition, the resulting MMN is seen to solidify and improve over time.

| **Subject** | **Gender** | **Age years** | **UBDRS PS** | **(*CLN*3SS)** | **Age of Vision Loss** | **Age of Seizures OS** | **Ambulatory** | **Age of Cognitive R** | **Medications** |  |  |  |  |
| --- | --- | --- | --- | --- | --- | --- | --- | --- | --- | --- | --- | --- | --- |
| 1 | Male | 28 | 61 | 3 | 6 yrs., 2 mon | 4 years | No | 10 yrs., 0 mon | Trazadone, Depakote |  |  |  |  |
| 2 | Male | 17 | 32 | 3 | 5 yrs., 6 mon | 6 years | No | 8 yrs., 6 mon | Lovastatin, Topomax, Abilify, Lexapro, Depakene, Nexium |  |  |  |  |
| 3 | Male | 19 | 14 | 2 | 5 yrs., 6 mon | 14 years | Yes | 7 yrs., 6 mon | Lovastatin, Ambien, Trazodone |  |  |  |  |
| 4 | Male | 20 | 40 | 2 | 5 yrs., 6 mon | 10 years | Yes | 5 yrs., 9 mon | Copaxone, Topomax, Lovastatin, |  |  |  |  |
| 5 | Female | 22 | 67 | 3 | 8 yrs., 0 mon | 9 years | No | 8 yrs., 3 mon | Trazadone |  |  |  |  |
| 6 | Male | 9 | 3 | 1 | 7 yrs., 10 mon | no | Yes | N/A | Lexapro, Lovastatin, Prilosec |  |  |  |  |
| 7 | Female | 24 | 42 | 2 | 5 yrs., 9 mon | U | Yes | 6 yrs., 9 mon | Lovastatin |  |  |  |  |
| 8 | Male | 13 | 1 | 1 | 6 yrs., 5 mon | no | Yes | N/A | Lovastatin |  |  |  |  |
| 9 | Female | 10 | 2 | 1 | 9 yrs., 3 mon | no | Yes | 11 yrs., 6 mon | none |  |  |  |  |
| 10 | Male | 20 | N/A | 1 | 7 yrs, 6 mon | no | Yes | 7 yrs., 4 mon | Lexapro |  |  |  |  |
| 11 | Female | 16 | 15 | 1 | 6 yrs, 0 mon | no | Yes | 6 yrs., 10 mon | Lexapro, Copaxone, Trazodone, |  |  |  |  |
| 12 | Female | 16 | N/A | U | 5 yrs., 6 mon | 2 years | Yes | 11 yrs., 2 mon | Depakene, Lactulose, Lovastatin, |  |  |  |  |
| 13 | Female | 17 | 2 | 1 | 5 yrs., 6 mon | N/A | Yes | N/A | Depakene, Lexapro,Lactulose |  |  |  |  |
| 14 | Male | 20 | N/A | 3 | 8 yrs., 0 mon | N/A | No | 8 yrs., 3 mon | none |  |  |  |  |
| 15 | Male | 20 | 58 | 3 | 4 yrs., 6 mon | 13 years | No | 5 yrs., 6 mon | N/A |  |  |  |  |
| 16 | Male | 10 | 8 | 2 | 4 yrs., 11 mon | 6 years | Yes | 5 yrs., 0 mon | Risperdol and Necon |  |  |  |  |
| 17 | Male | 6 | 2 | 1 | 5 yrs, 0 mon | N/A | Yes | 6 yrs., 0 mon | Depakote, Carintor, Artane, Copaxone |  |  |  |  |
| 18 | Female | 16 | 10 | 2 | 8 yrs., 11 mon | 10 years | Yes | 12 yrs., 6 mon | Depakene |  |  |  |  |
| 19 | Female | 14 | 45 | 3 | 6 yrs., 6 mon | 10 years | No | 6 yrs., 0 mon | none |  |  |  |  |
| 20 | Female | 14 | 1 | 1 | 12 yrs., 0 mon | N/A | Yes | N/A | Abilify, Lexapro, Desyrel |  |  |  |  |
| 21 | Female | 15 | 3 | 2 | 8 yrs., 0 mon | 11 years | Yes | 12 yrs., 9 mon | Diastat, Depakene, Carnitor |  |  |  |  |
| 22 | Female | 7 | 2 | 1 | 5 yrs., 3 mon | N/A | Yes | 1 yrs., 3 mon | Lexapro, Lamictal |  |  |  |  |
| 23 | Male | 8 | 3 | 2 | 6 yrs., 6 mon | 6 years | Yes | 6 yrs., 4 mon | Lexapro, Lamictal |  |  |  |  |
| 24 | Female | 15 | 60 | 3 | 6 yrs., 6 mon | 6 years | No | 8 yrs., 6 mon | Lexapro, Lamictal |  |  |  |  |
| 25 | Female | 13 | 13 | 2 | 5 yrs., 0 mon | 6 years | Yes | 10 yrs., 0 mon | Lexapro, Lamictal |  |  |  |  |

**Supplementary Table 1.** Clinical demographics of all enrolled participants with CLN3 disease. Data from four participants (Subjects 22-25) in in the shaded cells were excluded from EEG analysis. *CLN3*SS = *CLN3* Staging Score; Seizure OS = Seizure onset; Age of Cognitive R = Age of regression in years (yrs.) and months (mon); N/A = Not Available, U = undefined, UBDRS PS– Unified Batten Disease Rating Scale Physical subscale score.

|  |  |  |  |  |  |  |  |  |  |
| --- | --- | --- | --- | --- | --- | --- | --- | --- | --- |
|  | TD (n)=41 Mean Age 13.9±5.2 | | | CLN3 (n)=21 Mean Age 16.9±5.5 | | | TD mean of all accepted | CLN3 mean of all accepted |  |
|  |  |  |  |  |  |  |  |  |  |
|  |  |  |  |  |  |  |  |  |  |
|  | **450 SOA** | **900 SOA** | **1800 SOA** | **450 SOA** | **900 SOA** | **1800 SOA** | **Across Conditions** | **Across Conditions** |  |
| Avg. Accepted Standard trial ± SD | 767 ±58 | 742 ±90 | 737 ±115 | 716 ±97 | 722 ±132 | 616 ±192 | 749  ±61 | 684  ±99 |  |
| Avg. Accepted Deviant trial ± SD | 135 ±11 | 132 ±17 | 129 ±20 | 125 ±17 | 128 ±24 | 107 ±33 | 132  ±11 | 120  ±17 |  |

**Supplementary Table 2.** Numbers of trial included in the analysis across condition in controls and in participants with CLN*3* disease.
